# Smart Sensor-Driven Gait Rehabilitation Walker Using Machine Learning for Predictive Home-Based Therapy

**DOI:** 10.3390/s26082547

**Published:** 2026-04-21

**Authors:** Gokul Manavalan, Yuval Arnon, A. N. Nithyaa, Shlomi Arnon

**Affiliations:** 1 Department of Biomedical Engineering, Rajalakshmi Engineering College, Chennai 602105, India; 2 Electrical and Computer Engineering Department, Ben-Gurion University of the Negev, Beer Sheva 8410501, Israel; 3 Medical School for International Health, Ben-Gurion University of the Negev, Beer Sheva 8410501, Israel; arnoy@post.bgu.ac.il

**Keywords:** abnormal gait, smart rehabilitation walker, home rehabilitation, haptic feedback, force symmetry, Gaussian Process Regression

## Abstract

**Highlights:**

**What are the main findings?**

- A modular smart rehabilitation walker provides real-time haptic symmetry feedback.
- Multimodal sensing (FSR, sEMG, IMU) enables continuous gait monitoring.
- The Force Symmetry Index decreased by an average of 79.26% during a 15-day rehabilitation study.
- EMG activation significantly increased (ΔEMG = 4.28, t(9) = 13.58, *p* < 0.001).

**What are the implications of the main findings?**

- The system supports more balanced gait and improved neuromuscular activation during rehabilitation.
- Gaussian Process Regression enables uncertainty-aware predictions for personalized therapy.
- The analytical pipeline demonstrates generalizability through validation on an external ALS gait dataset.

**Abstract:**

Abnormal gait associated with neuromuscular and musculoskeletal disorders represents a growing clinical burden, particularly in aging populations. This study presents a modular, low-cost Smart Rehabilitation Walker (SRW) that integrates multimodal sensing and real-time haptic feedback to enable simultaneous gait monitoring and corrective intervention in both clinical and home environments. The system combines force-sensing resistors for bilateral load symmetry assessment, inertial measurement units for fall detection, and surface electromyography (sEMG) for neuromuscular activity monitoring within a closed-loop assistive feedback architecture. A 15-day pilot study involving ten individuals with rheumatoid arthritis and clinically observed neurological gait abnormalities demonstrated measurable improvements in gait biomechanics. The Force Symmetry Index (FSI), calculated using the Robinson symmetry metric, decreased from an average of 0.9691 to 0.2019, corresponding to a 79.26% average reduction in inter-limb load asymmetry. Concurrently, sEMG measurements showed a substantial increase in neuromuscular activation (ΔEMG = 4.28), with statistical analysis confirming a significant improvement across participants (paired *t*-test: t(9) = 13.58, *p* < 0.001). To model rehabilitation trajectories, a nonlinear predictive framework based on Gaussian Process Regression achieved high predictive accuracy (R^2^ ≈ 0.9, with a mean RMSE of 0.0385), while providing uncertainty-aware trend estimation. Validation using an independent amyotrophic lateral sclerosis gait dataset further demonstrated the transferability of the analytical pipeline. These results highlight the potential of sensor-enabled assistive walkers as scalable platforms for quantitative gait rehabilitation, adaptive feedback, and long-term mobility monitoring.

## 1. Introduction

Abnormal gait patterns are widely observed in neuromuscular, musculoskeletal, and neurological disorders and represent a major contributor to reduced mobility, loss of independence, and elevated fall risk [1,2,3]. Such impairments may occur transiently, as in orthopedic injury or post-operative recovery, or persist chronically in conditions including rheumatoid arthritis (RA), post-stroke hemiplegia, demyelinating disorders, and neurodegenerative diseases [2,4,5,6]. Despite their diverse etiologies, these disorders frequently produce similar functional manifestations such as gait asymmetry, postural instability, altered limb loading, and inefficient force distribution during ambulation [7,8].

Post-stroke hemiplegia is commonly regarded as a canonical model of asymmetric gait impairment, characterized by unilateral weakness, impaired balance, and compensatory locomotor strategies [2,8,9]. Comparable biomechanical and neuromotor deficits are also observed in individuals with chronic inflammatory joint diseases such as RA, as well as in neurological conditions associated with spasticity, fatigue, and impaired proprioception [7,10]. Without appropriate rehabilitation support, these impairments may progress toward maladaptive gait patterns, joint overloading, and increased fall susceptibility [7].

Epidemiological studies further highlight the scale of this challenge. Rheumatoid arthritis alone contributes to significant global morbidity and functional disability, while neurological gait disorders associated with degenerative and demyelinating conditions continue to increase with aging populations [10]. These trends emphasize the need for accessible and scalable rehabilitation technologies capable of supporting long-term mobility outside traditional clinical settings [11,12].

Conventional walkers and assistive mobility aids provide mechanical stability during ambulation but typically lack real-time monitoring, adaptive feedback, or quantitative assessment of user–device interaction [13,14]. In particular, asymmetric force application on walker handlebars—commonly observed in individuals with unilateral motor impairment—remains largely unquantified despite its association with fatigue, postural misalignment, and secondary musculoskeletal strain [14,15,16,17].

Importantly, upper-limb force asymmetry during walker-assisted ambulation is closely associated with underlying gait impairments. Individuals with unilateral weakness or neuromotor deficits often compensate by disproportionately loading one side of the walker, resulting in uneven weight distribution, postural misalignment, and inefficient gait mechanics.

Load asymmetry and excessive upper extremity (UE) force impair gait and balance, increasing the risk of instability and injury. During walker-assisted ambulation, users often exceed recommended force limits, leading to asymmetrical loading and increased stress on the upper body. Feedback-based interventions improve force modulation and reduce muscle overactivation. Biomechanical analyses further indicate that the shoulder experiences the highest load during walker use, making it particularly vulnerable to overuse. These findings highlight the need for improved force control, load symmetry, and ergonomic assistive device design [18,19].

Therefore, monitoring and correcting force asymmetry at the walker interface provides a practical and quantifiable proxy for improving overall gait performance. By promoting balanced upper-limb loading, it is possible to indirectly support more symmetrical lower-limb movement patterns and enhance functional mobility during rehabilitation. Advanced robotic rehabilitation systems can provide effective training but are often limited by high cost, infrastructure requirements, and restricted suitability for home-based deployment [14,15,16,17].

To address these limitations, this work presents a modular Smart Rehabilitation Walker (SRW) designed to support continuous gait monitoring and corrective feedback in home and community environments. The system integrates open-source embedded hardware with multimodal sensing, including force-sensing resistors for load symmetry monitoring, inertial measurement units for fall detection, and surface electromyography for neuromuscular activity analysis. A closed-loop haptic feedback mechanism is implemented to encourage voluntary correction of asymmetric upper-limb force during assisted walking.

Recent advances in rehabilitation technologies have emphasized the development of wearable and adaptive assistive systems for real-world mobility support. For example, Wearable technologies for assisted mobility in the real world highlights the integration of multimodal sensing, including physiological and environmental signals, to enable human-in-the-loop adaptive assistance in unconstrained environments [20]. Similarly, Reconfigurable Exomuscle System Employing Parameter Tuning to Assist Hip Flexion or Ankle Plantarflexion presents a reconfigurable exomuscle system capable of dynamically adjusting assistive parameters to reduce metabolic cost during walking [21]. While these systems demonstrate significant advances in adaptive actuation and wearable assistance, their complexity and cost may limit large-scale deployment in home-based rehabilitation settings. In contrast, the present study focuses on a modular SRW that combines multimodal sensing with real-time haptic feedback and interpretable predictive modeling, providing a scalable and cost-effective solution for individualized gait rehabilitation in real-world environments.

In addition to hardware development, a data-driven analytical framework is introduced to quantify rehabilitation progress. Force-distribution signals obtained from the walker are analyzed using a nonlinear predictive model based on Gaussian Process Regression, enabling individualized modeling of rehabilitation trajectories while incorporating uncertainty estimation. To further examine the robustness of the analytical pipeline beyond the pilot study cohort, the framework is additionally evaluated using an independent open-source gait dataset associated with Amyotrophic Lateral Sclerosis. By integrating sensor-enabled assistive hardware with predictive data analytics, the proposed system aims to bridge the gap between conventional mobility aids and intelligent rehabilitation platforms. The smart rehabilitation walker provides a scalable and interpretable approach for monitoring gait symmetry, supporting corrective intervention, and facilitating home-based rehabilitation across a range of mobility impairments.

A 15-day pilot study involving ten individuals with rheumatoid arthritis and neurological gait abnormalities was conducted to evaluate the system. Quantitative analysis demonstrated a substantial improvement in gait symmetry, with the Force Symmetry Index decreasing from 0.953 to 0.153 (83.9% reduction for subject 1), alongside a significant increase in neuromuscular activation. These results demonstrate the potential of the proposed system for closed-loop gait rehabilitation and objective monitoring of functional recovery.

### 1.1. Related Work in Abnormal Gait Measurement

Over the past decade, abnormal gait assessment has evolved from laboratory-based biomechanical analysis toward scalable sensing and data-driven monitoring approaches. Contemporary research can be broadly categorized into vision-based systems, wearable sensing methods, structural vibration monitoring, and hybrid multimodal frameworks.

#### 1.1.1. Vision-Based Methods

Vision-based gait analysis commonly relies on optical motion capture or computer vision techniques. Markerless video-based systems utilize standard RGB cameras to extract gait kinematics without specialized markers. For example, large annotated video datasets have enabled deep learning models to classify abnormal gait patterns with high accuracy [22]. Self-supervised frameworks such as FSGait further reduce dependence on manually labeled data by learning fine-grained spatiotemporal features directly from video sequences [23]. These approaches offer scalability and non-invasive monitoring; however, their performance is often sensitive to environmental factors such as illumination variations, occlusions, and background complexity.

In contrast, marker-based optical motion capture systems remain the gold standard for biomechanical gait analysis. These systems employ reflective markers and multi-camera setups to reconstruct high-resolution three-dimensional skeletal motion [24]. Although they provide highly accurate kinematic measurements and detailed biomechanical insight, their deployment typically requires specialized laboratory infrastructure, expensive instrumentation, and trained personnel, which limits their accessibility for routine or home-based monitoring.

#### 1.1.2. Wearable Sensor-Based Methods

Wearable sensing technologies have emerged as a practical alternative for continuous gait monitoring in real-world environments. Inertial measurement units (IMUs) are widely used to capture joint kinematics and gait dynamics through accelerometer and gyroscope signals. Machine learning algorithms applied to IMU-derived features have demonstrated promising performance in identifying gait abnormalities associated with orthopedic and neurological disorders [25]. Despite their portability and cost-effectiveness, IMU-based systems remain sensitive to sensor placement and alignment, which can affect measurement reliability.

Surface electromyography (sEMG) sensors provide complementary information by measuring neuromuscular activity during locomotion. Recent studies have applied deep learning architectures, including LSTM and BiLSTM models, to sEMG signals for predicting fall risk and detecting abnormal motor patterns [26]. While these methods offer valuable physiological insights into muscle activation and coordination, sEMG measurements are susceptible to motion artifacts and electrode displacement, requiring careful sensor placement and calibration.

#### 1.1.3. Structural Vibration-Based Methods

Structural vibration monitoring represents an alternative indirect approach to gait analysis. In such systems, floor-mounted sensors detect vibrations generated by footstep impacts during walking. Frequency-domain analysis of these vibration signals can reveal distinctive characteristics associated with abnormal gait patterns [27,28]. These systems enable passive and unobtrusive monitoring; however, their implementation typically requires specialized instrumented environments and may be affected by ambient noise or environmental disturbances.

#### 1.1.4. Hybrid Multimodal and Data-Augmented Methods

Hybrid approaches combine multiple sensing modalities with advanced machine learning techniques to improve robustness and generalization. For example, multimodal frameworks integrating temporal deep learning architectures with generative adversarial networks (GANs) have been proposed to augment limited datasets and enhance classification performance for rare gait disorders [29]. While such methods can achieve high predictive accuracy, their complexity often introduces challenges related to computational cost, model interpretability, and clinical translation.

#### 1.1.5. Positioning of the Proposed Approach

Despite significant progress in sensing and machine learning techniques, many existing systems remain constrained by laboratory infrastructure, complex instrumentation, or limited real-time rehabilitation capabilities. In contrast, the system proposed in this work focuses on a portable and assistive sensing platform capable of both monitoring and corrective intervention during walking. The proposed smart rehabilitation walker integrates force-sensing resistors, inertial measurement units, and surface electromyography sensors to capture multimodal information related to balance, load distribution, and neuromuscular activity. Unlike purely observational gait analysis systems, the walker incorporates a closed-loop haptic feedback mechanism to encourage voluntary correction of asymmetric force application during assisted ambulation. Furthermore, rehabilitation progress is modeled using a nonlinear predictive framework based on Gaussian Process Regression, enabling individualized trend analysis with uncertainty estimation. A comparative summary of representative gait monitoring approaches is provided in Table 1.

### 1.2. Related Work in Real-Time Feedback for Gait Rehabilitation

The importance of adaptive feedback mechanisms is supported by prior studies. Zhang et al. demonstrated high-accuracy fatigue detection in older adults using IMU and plantar force data [30]. However, their system relied on treadmill-based recordings under controlled laboratory conditions, limiting its applicability to real-world rehabilitation scenarios. In contrast, the SRW proposed here enables continuous monitoring during free-living walking conditions, making it more suitable for long-term home rehabilitation. Similarly, Cleland et al. used wearable sensors to analyze post-stroke gait and identified predictors of walking speed and endurance [9]. While their system provided valuable diagnostic insights, it did not incorporate real-time corrective feedback. The SRW developed in this study addresses this limitation by delivering instantaneous haptic cues that guide users toward symmetrical arm loading, thereby transforming passive monitoring into an active rehabilitation intervention.

The integration of bilateral haptic feedback also aligns with the concept of arm–leg coupling in locomotor control, which has been highlighted as an important mechanism in gait coordination [31]. By delivering vibrotactile cues to both upper limbs while simultaneously monitoring EMG activity, the proposed system enables direct observation of the relationship between neuromuscular activation and biomechanical symmetry. This multimodal approach provides richer information than feedback systems relying on a single sensing modality.

From a data analytics perspective, the use of Gaussian Process Regression addresses the need for transparent and interpretable predictive modeling in rehabilitation systems. Previous work by Yentes and colleagues emphasized the importance of methodological rigor and parameter transparency in gait time-series analysis [32]. The GPR model employed in this study satisfies these requirements by providing probabilistic predictions with confidence intervals, allowing clinicians to interpret rehabilitation trajectories with quantified uncertainty. Complementary research by Franck et al. demonstrated the value of Bayesian statistical modeling for gait analysis [33]. Although Bayesian approaches offer rigorous uncertainty quantification, they often require complex computational frameworks and specialized expertise. The GPR implementation used here provides a practical compromise, enabling interpretable predictions while maintaining computational efficiency suitable for real-time applications.

Recent work in rehabilitation robotics further highlights the advantages of multi-sensor integration. Lee et al. demonstrated that lower-limb kinematics could predict rectus femoris hyperreflexia in post-stroke patients using a powered orthosis combined with biomechanical simulations [34]. While their approach achieved high accuracy, it required detailed kinematic modeling and muscle simulations that are difficult to deploy outside specialized laboratories. In contrast, the SRW developed in this work achieves predictive monitoring using integrated physiological and force sensors, reducing the need for complex biomechanical modeling and enabling practical home-based deployment. A structured comparison of the proposed system with representative recent gait rehabilitation and monitoring approaches is summarized in Table 2. The comparison highlights that while many existing solutions focus on isolated sensing modalities or single clinical populations, the present SRW integrates multimodal sensing (EMG, IMU, and force sensors) with real-time haptic feedback and interpretable predictive modeling. This integrated architecture enables simultaneous monitoring, intervention, and rehabilitation assessment within a single portable platform designed for home-based use.

## 2. Materials and Methods

The proposed framework was developed to identify, characterize, and support abnormal gait patterns in individuals with musculoskeletal and neurological mobility impairments. The system combines a sensor-integrated assistive walker with data-driven predictive modeling to enable both gait monitoring and rehabilitation support in home environments. The experimental framework consists of two main phases. Phase I involves multimodal sensor-based gait screening to capture biomechanical and neuromuscular signals during assisted walking. These signals are processed to quantify gait asymmetry and user–walker interaction patterns. Phase II involves a Smart Rehabilitation Walker protocol in which real-time feedback is provided to encourage voluntary correction of asymmetric force distribution and improve balance control. The full clinical workflow for screening, assessment, and rehabilitation procedures is provided in Appendix A, while this section focuses on the engineering design, sensing architecture, predictive modeling framework, and pilot study protocol used to evaluate the system.

### 2.1. Smart Rehabilitation Walker Design

A Smart Rehabilitation Walker (SRW) was developed as a modular assistive platform capable of monitoring gait dynamics and providing real-time feedback during ambulation. Conventional walkers typically provide mechanical stability but lack sensing capabilities, adaptive feedback mechanisms, or quantitative monitoring of user–device interaction [13,14]. The proposed SRW addresses these limitations by integrating sensing, feedback, and predictive modeling components into a portable rehabilitation device.

The prototype walker used in this study had a fixed height of 81 cm, which lies within the typical adjustment range (81–102 cm) of commercially available walkers [35]. The finalized design is illustrated in Supplementary Materials Figure S1.

However, it is acknowledged that this configuration may not be optimal for all users, particularly individuals shorter than the intended height range. Previous studies have demonstrated that improper walker height can influence upper-limb loading, posture, and joint stress during assisted ambulation, potentially affecting gait mechanics and user comfort [36]. As a result, the use of a fixed-height walker may introduce variability in ergonomic conditions across participants. Despite this limitation, all participants were able to safely complete the study protocol, and no adverse effects were observed. Since the analysis focuses on within-subject changes in force symmetry and neuromuscular activity, the impact of absolute height mismatch is partially mitigated. Nevertheless, future work will incorporate adjustable walker configurations to ensure optimal ergonomic alignment and improve usability across a broader range of users.

**Figure 1:**
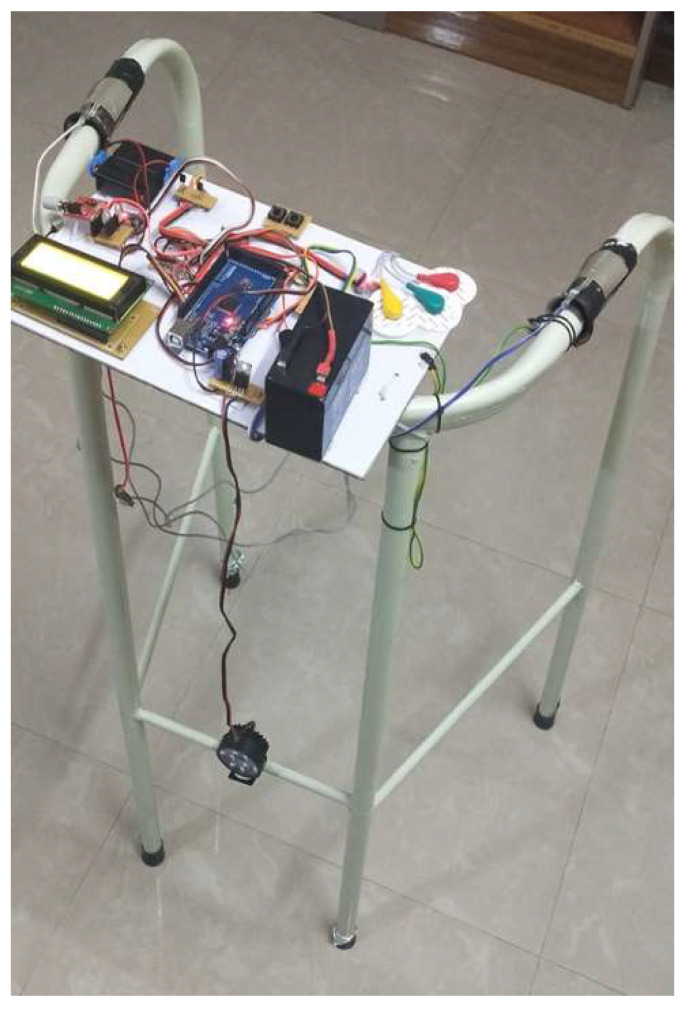
Final Assembled Smart Rehabilitation Walker with Embedded Modules (Front View): Photographic depiction of the operational Smart Rehabilitation Walker with integrated sensing, actuation, and wireless communication systems. The design ensures modular placement for stability, accessibility, and compactness.

The frame structure was fabricated using mild steel tubing to provide mechanical durability while maintaining manageable weight. The structure was coated with an aluminum layer to enhance corrosion resistance and structural longevity. The complete system, including sensing modules, microcontroller, and communication components, weighs approximately 3.1 kg, which falls within the typical range of commercial walker devices (2.5–5 kg) [37]. The modular architecture allows the sensing and processing units to be integrated without significantly increasing the physical burden on the user. Figure 1 and Supplementary Materials Figure S2 illustrate the assembled SRW prototype with integrated sensing modules and communication components. The design ensures stable sensor placement, compact electronics integration, and unobstructed hand support surfaces for the user.

### 2.2. Multimodal Sensor Modules

The SRW incorporates multiple sensing modules to capture biomechanical and physiological signals associated with gait dynamics. These modules enable monitoring of force distribution, walker orientation, and neuromuscular activity during assisted walking. All sensors are interfaced with a microcontroller platform responsible for signal acquisition, preprocessing, and feedback control.

(a) Balance Monitoring Module: To monitor asymmetric force distribution during walker-assisted ambulation, two Force-Sensitive Resistors (FSRs) were embedded within the walker handlebars. These sensors measure the vertical force exerted by the user’s hands. Each FSR produces an analog voltage proportional to the applied pressure, which is digitized using an Arduino-based microcontroller with a 10-bit analog-to-digital converter (ADC), yielding values in the range 0 ≤ V_ADC ≤ 1023 [38,39]. Prior to system integration, the FSRs were calibrated using known reference loads applied via a commercial weighing scale, enabling a mapping between sensor output and applied force. Real-time force symmetry was evaluated by comparing left and right handle forces [38,39].

When the force difference exceeded a predefined threshold, miniature vibration motors (VM0610A3.0, EKULIT Elektrotechnik Karl-Heinz Mauz GmbH, Ostfildern, Germany) mounted on the handles were activated [40,41]. In this study, haptic feedback is defined as a vibrotactile stimulus delivered through these motors to communicate force asymmetry to the user [42]. Although haptic interaction can include multiple modalities, vibration-based feedback provides an effective and perceptible cue for corrective action in assistive systems [43,44]. The vibration motors were configured with a frequency of 100 Hz, amplitude of 1.5 g, and duration of 300 ms, selected based on prior studies on human vibrotactile perception and comfort [45,46]. Pilot testing confirmed that these stimuli were clearly perceivable without causing discomfort.

The asymmetry threshold for triggering haptic feedback was determined during pilot testing using data collected from five subjects. Force asymmetry values were recorded across 100 instances under varying load conditions, and the threshold was defined based on the average observed asymmetry. This approach ensured that the threshold reflected typical user behavior in realistic usage scenarios. A universal threshold was applied to all participants to maintain consistency and simplify system deployment across individuals with varying physical characteristics. While this approach enables standardized evaluation, it does not account for subject-specific variability. Future work will investigate adaptive or individualized thresholding strategies to further enhance personalization and sensitivity of the feedback mechanism.

(b) Fall detection Module: A three-axis accelerometer was integrated into the walker structure to monitor its orientation and detect instability. The accelerometer measures linear acceleration along three orthogonal axes, defined as a = (ax, ay, az), where each component represents acceleration along the respective axis. Walker orientation was continuously monitored, and tilt angles exceeding predefined thresholds were interpreted as potential tipping events or unsafe walker positioning [47,48]. Upon detection, an auditory alert was generated using an integrated buzzer to notify nearby individuals or caregivers. It is important to note that this module detects walker instability, which may increase the risk of a user fall, but does not directly detect or predict actual falls. Therefore, the system should be considered as providing an additional safety layer through early warning of unsafe conditions, rather than a direct fall detection mechanism.
(c) sEMG Monitoring Module: To capture neuromuscular activity during assisted ambulation, a surface electromyography (sEMG) module was integrated into the system. Surface electrodes were placed over the biceps brachii muscle following standard sEMG guidelines, with the active electrode positioned on the muscle belly and the reference electrode near the elbow joint. The recorded signal reflects muscle activation and is expressed in millivolts [41,49,50]. Due to the susceptibility of sEMG signals to noise sources such as motion artifacts, electrode–skin impedance variations, and environmental interference, a preprocessing pipeline was implemented to enhance signal quality. A 4th-order digital band-pass filter (30–500 Hz) was applied to remove low-frequency drift and high-frequency noise [51]. Subsequently, wavelet-based denoising was used to further suppress residual noise, including artifacts introduced during Bluetooth transmission [52]. These preprocessing steps improve the signal-to-noise ratio and ensure reliable extraction of muscle activation patterns. The processed signals were then used to analyze muscle engagement and variations during walker-assisted ambulation. A detailed description and corresponding figure of the sEMG signal processing are provided in Appendix B.
(d) Environmental Assistance and Communication: To improve usability in low-light environments, a light-dependent resistor (LDR) sensor was integrated into the walker system. The LDR monitors ambient illumination levels and activates auxiliary lighting when environmental brightness falls below a predefined threshold. This feature enhances user safety during nighttime or low-visibility conditions. Wireless data transmission was enabled through a Bluetooth communication module, allowing real-time streaming of sensor data to external devices for monitoring, data logging, or cloud-based analysis.

### 2.3. Predictive Modeling Using Gaussian Process Regression

To analyze force signals recorded from the FSR sensors and model rehabilitation progress over time, a predictive framework based on Gaussian Process Regression was implemented. Gaussian Process Regression (GPR) is a Bayesian non-parametric modeling approach that provides both predictive estimates and uncertainty quantification, making it particularly suitable for clinical datasets that may be limited or noisy [53,54].

#### 2.3.1. Gaussian Process Model

A Gaussian Process defines a distribution over functions and is fully specified by a mean function $m\left(x\right)$ and a covariance function $k\left(x,x^{\prime }\right)$ [53,54,55]. The model can be expressed as:

(1) $$y\left(x\right)\sim GP\left(m\left(x\right),k\left(x,x^{\prime }\right)\right)$$

where x represents the input variable and $y\left(x\right)\;$ denotes the predicted output. In this work, the mean function was modeled as a constant value estimated from the training data.

#### 2.3.2. Kernel Function

The covariance structure of the GP model was defined using a Radial Basis Function (RBF) kernel, expressed as [56]:

(2) $$k\left(x,x^{\prime }\right)=\sigma_{f}^{2}\exp\left(-\frac{{\left(x-x^{\prime }\right)}^{2}}{2l^{2}}\right)$$

where $\sigma_{f}^{2}$ is the signal variance and l is the characteristic length-scale controlling smoothness [54]. The RBF kernel assumes smooth and continuous variation in the underlying function, which is appropriate for modeling gradual rehabilitation trends over time.

#### 2.3.3. Inference and Prediction

Given training inputs x and observed outputs y, the predictive distribution at a new test point $x^{\prime }$ is Gaussian, with mean and variance [57]:

(3) $$\mu \left(x^{\prime }\right)=k^{T}{\left(K+\sigma_{n}^{2}I\right)}^{-1}y$$

(4) $$\sigma^{2}\left(x^{\prime }\right)=k_{**}-k^{T}{\left(K+\sigma_{n}^{2}I\right)}^{-1}k$$

Here, K is the kernel matrix evaluated on the training data, k is the kernel vector between $x^{\prime }$ and training inputs, $k_{**}=\;\left(x^{\prime },x^{\prime }\right)$, and $\sigma_{n}^{2}$ is the observation noise variance [55,57]. The model hyperparameters $\theta =\left\{\sigma_{f}^{2},l,\sigma_{n}^{2}\right\}$ are optimized by maximizing the log marginal likelihood:

(5) $$\mathrm{log p}\left(\left.y\right|x,\theta \right)=-\frac{1}{2}y^{T}{\left(K+\sigma_{n}^{2}I\right)}^{-1}y-\frac{1}{2}log|K+\sigma_{n}^{2}I|-\frac{n}{2}log(2\pi )$$

where n is the number of training samples, and $\mathrm{log p}\left(\left.y\right|x,\theta \right)$ represents the log-likelihood of observing the data under the GP model.

#### 2.3.4. Application to Gait Force Analysis

The GPR model was applied to estimate and predict FSR resistance values during walker-assisted ambulation. The input variable *x* corresponds to the time index of the sensor measurement, while the output variable *y* represents the measured FSR resistance associated with user-applied handlebar force. By training on pooled data from all participants, the model captures shared biomechanical patterns while minimizing overfitting to individual subjects. Model performance was evaluated using held-out test data, ensuring that predictions were assessed on previously unseen samples. This generalized modeling approach supports trend analysis across the RA population studied, while future work will focus on personalized models to further improve predictive accuracy for individual patients. The GPR framework additionally provides confidence intervals, enabling uncertainty-aware prediction of rehabilitation trajectories. It should be noted that this approach evaluates generalization within the observed cohort rather than across entirely unseen subjects.

#### 2.3.5. Dataset and Validation

The model was trained on longitudinal FSR data collected from 10 participants over a 15-day rehabilitation period, comprising 1200 time-series samples per sensor channel. For each participant, 80% of the data were used for training, while the remaining 20% were reserved as a held-out test set. This split ensures that evaluation is performed on previously unseen samples, although data from the same subjects are present in both sets.

In addition, 5-fold cross-validation was performed on the training data to optimize hyperparameters and reduce overfitting. Pooling data across all participants enables the model to capture shared trends in force distribution across individuals with varying characteristics. However, this approach does not represent subject-independent validation, and future work will explore cross-subject generalization and personalized modelling strategies. A detailed summary of the dataset, training/test splits, and model performance metrics is provided in Table A1 in Appendix C.

### 2.4. Study Participants and Clinical Relevance

To evaluate the real-world efficacy of the proposed SRW system, a 15-day pilot study was conducted involving ten voluntary participants presenting with clinically established chronic gait abnormalities. All participants had a confirmed diagnosis of rheumatoid arthritis (RA), a progressive autoimmune disorder known to compromise joint stability, posture, and locomotor function. In addition to RA-related impairments, several participants exhibited neurological gait features commonly associated with demyelinating disorders, such as spasticity, proprioceptive deficits, muscle fatigue, intermittent balance instability, and altered foot clearance. Some also demonstrated mild residual effects from a prior stroke, including subtle unilateral weakness, occasional coordination challenges, and mild balance difficulties, all of which subtly influenced their mobility. While formal neurological confirmation of multiple sclerosis (MS) was not uniformly available, these features were clinically observed and functionally documented during supervised walking sessions.

Subject 1, in particular, exhibited a pronounced mixed-impairment phenotype, combining antalgic gait patterns characteristic of advanced RA with marked neurological gait disturbances, such as spastic limb motion, intermittent foot drop, and residual mild effects from a previous stroke. This presentation provided a valuable stress-test scenario for evaluating the adaptive capabilities of the SRW’s real-time haptic feedback and EMG-based neuromuscular monitoring modules. Table 3 summarizes the demographic and functional characteristics of the study participants. While rheumatoid arthritis served as the primary confirmed diagnosis across the cohort, the observed neurological gait phenotypes highlight the broader applicability of the proposed SRW to mobility impairments characterized by postural asymmetry and neuromotor instability, including demyelinating disorders, post-stroke hemiplegia, Parkinsonian gait, spinal cord injury, and orthopedic rehabilitation contexts.

The study duration of 15 days was intentionally selected as a pilot evaluation window to investigate short-term user adaptation, system usability, and immediate therapeutic response to the proposed SRW. Similar short-duration protocols have been widely adopted in rehabilitation robotics to assess early-stage gait adaptation and functional improvement. Recent evidence further indicates that measurable improvements in gait and balance can occur within approximately 10–15 training sessions, supporting the use of such timeframes for pilot validation [58,59]. Within this controlled duration, the study enabled close monitoring of patient–device interaction, early-stage neuromuscular response, and potential ergonomic concerns. No adverse effects or user discomfort were reported during the intervention period. Nevertheless, extended longitudinal studies are required to comprehensively evaluate long-term ergonomic safety, musculoskeletal adaptation, and sustained rehabilitation outcomes.

### 2.5. Multimodal System Integration and Functional Validation

**Figure 2:**
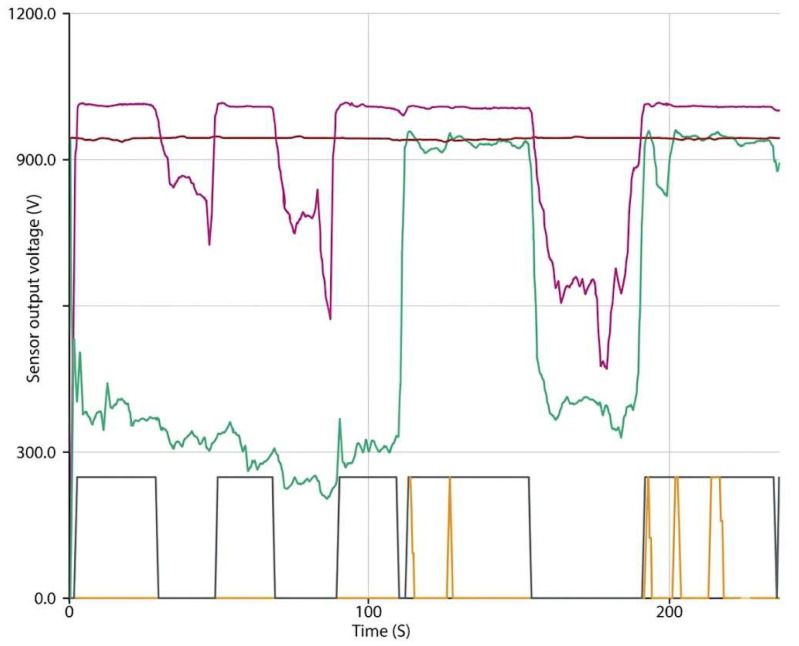
Real-Time Force Distribution and Haptic Feedback Mapping: Arduino serial plotter output showing FSR1 (green) and FSR2 (pink) resistance data alongside haptic feedback activation signals—right side (orange), left side (gray)—captured during live walker use. Asymmetry-triggered feedback loops demonstrate closed-loop correction in real time. The red line represents the LDR in the SRW at its baseline; it exhibits a peak when exposed to a dusky environment, reflecting the change in resistance values.

The developed smart rehabilitation walker integrates force sensing, surface electromyography (sEMG), inertial sensing, vibrotactile feedback, fall detection, and lighting modules within a closed-loop rehabilitation framework. Sensor signals are processed in real time by the onboard microcontroller, and outputs are simultaneously transmitted through Bluetooth for remote monitoring while also being displayed locally on an LCD interface. The force sensing subsystem uses bilateral force-sensitive resistors (FSRs) mounted on the walker handles to measure upper-limb loading distribution during locomotion. When the inter-arm force difference exceeded the predefined asymmetry threshold, the system triggered vibrotactile feedback to guide corrective posture adjustment. Figure 2 presents representative real-time force signals and haptic feedback activation, illustrating the closed-loop corrective mechanism. Periods of asymmetric loading between the right (FSR1) and left (FSR2) arms correspond to activation of the vibrotactile motors, confirming successful detection and feedback triggering. The average response latency of the feedback loop was measured at <120 ms, ensuring real-time correction during walking.

**Figure 3:**
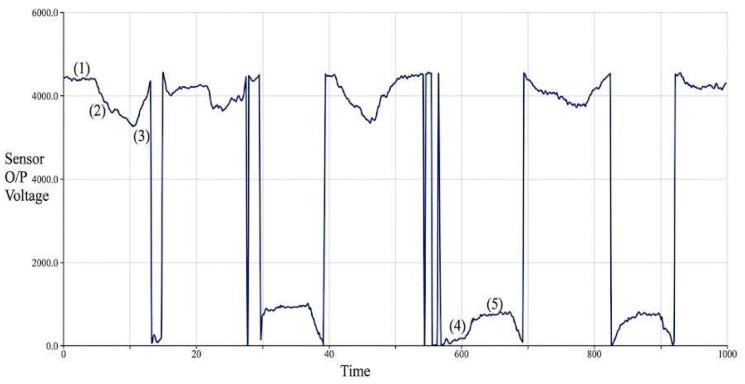
Acceleration signal pattern captured via the Arduino serial plotter, demonstrating the effectiveness of the fall detection algorithm. The tri-axial accelerometer distinctly identifies multiple positional states of the walker: upright (State 1), inclined (States 2 and 4), and falling (States 3 and 5). These transitions validate the algorithm’s capability for early fall prediction and proactive stability management.

The fall detection module was evaluated using controlled orientation tests. Figure 3 shows the tri-axial acceleration profiles captured during three walker states: upright, inclined, and falling. Distinct threshold crossings in the acceleration signals enabled reliable discrimination between these states. The algorithm correctly identified simulated fall events with 100% detection accuracy across repeated trials, demonstrating reliable stability monitoring.

**Figure 4:**
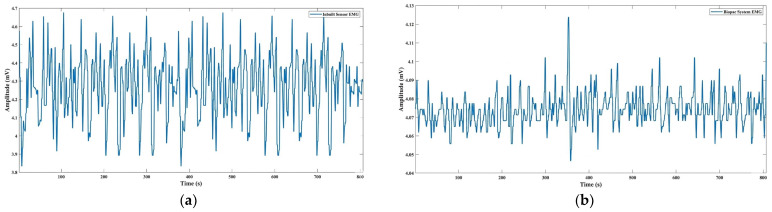
Validation of Embedded EMG Acquisition Module; (**a**): Raw Surface EMG Signal Acquisition Using Embedded sEMG Sensor: Captured surface electromyography (sEMG) signals reflect baseline muscle activation of the upper limb during walker-assisted locomotion; (**b**): Raw EMG Signal Acquisition Using Biopac System: sEMG recordings obtained from a Biopac MP36 system for comparative signal validation, highlighting consistency with the embedded EMG module in amplitude and waveform morphology.

To validate the embedded EMG acquisition module, signals recorded from the custom hardware were compared with recordings from a commercial Biopac MP36 system. Figure 4 illustrates representative signal traces from both systems. The root-mean-square (RMS) deviation between the two signals was ΔRMS = 0.2, indicating high agreement between the embedded system and the clinical reference device.

Additional operational interfaces were also evaluated. Bluetooth connectivity enabled real-time wireless data streaming, complemented by an integrated LCD for immediate user feedback, as shown in Supplementary Figures S3 and S4. To assess the lighting module, tests were conducted under low ambient illumination, confirming significantly enhanced visibility for night-time operation (Supplementary Figure S5). Implementation details—including electrode placement and the user interfaces—are documented in Supplementary Figure S6. Collectively, these results confirm that the proposed system successfully integrates multi-sensor data acquisition, real-time signal processing, and adaptive feedback control within a portable rehabilitation platform.

### 2.6. Force Symmetry Index (FSI) Estimation

To quantitatively evaluate upper limb symmetry improvement, a Force Symmetry Index (*FSI*) was computed using the widely adopted symmetry metric introduced by R. O. Robinson and colleagues [60,61].

(6) $$FSI=\frac{\left|F_{R}-F_{L}\right|}{F_{R}+F_{L}}$$

where $F_{R}$ and $F_{L}$ represent right- and left-hand force measurements, respectively. FSI values range from 0 (perfect symmetry) to 1 (maximum asymmetry), providing a normalized measure of force imbalance between the right and left hands [60,61].

## 3. Results

### 3.1. Walker-Assisted Gait Therapy Evaluation

To evaluate therapeutic performance, ten participants with varying gait abnormalities completed a 15-day walker-assisted rehabilitation pilot protocol, consistent with short-duration intervention frameworks commonly used in rehabilitation robotics. Each participant performed daily walking sessions of approximately 80 m, during which real-time vibrotactile feedback was provided upon detection of asymmetric force application. This duration enabled systematic observation of user adaptation, gait correction behavior, and neuromuscular engagement during early-stage rehabilitation. While the results demonstrate feasibility and short-term therapeutic benefits, the study does not address long-term ergonomic safety or sustained clinical outcomes, which remain important directions for future investigation. All images in the main manuscript and Supplementary Materials regarding tested subjects are fully deidentified and are used solely to illustrate posture progression. Written consent to use these figures was obtained from the respective individuals, and Institutional Review Board (IRB) approval was granted; full details of ethical clearance are provided in the footnotes.

**Figure 5:**
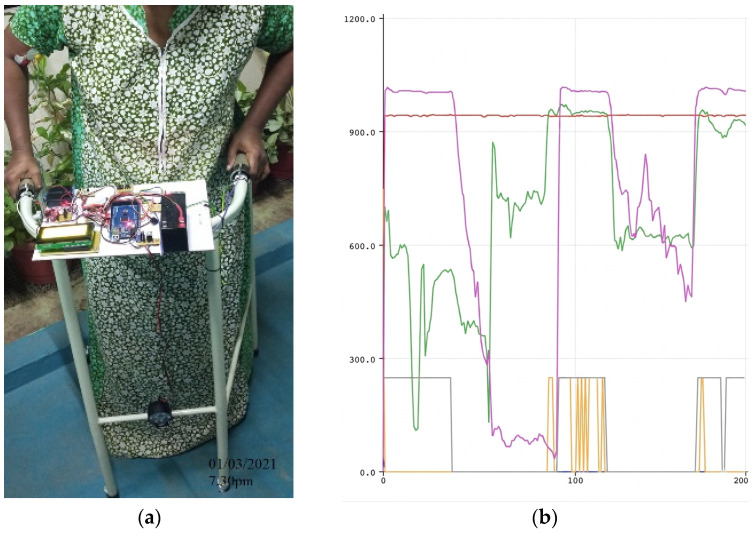
Gait Pattern and Posture Assessment on Day 1: (**a**) Subject 1’s baseline posture captured during initial trial; (**b**) Corresponding FSR signal plot reveals asymmetric upper-limb loading and irregular force compensation with Force Symmetry Index (FSI) of 0.953.

**Figure 6:**
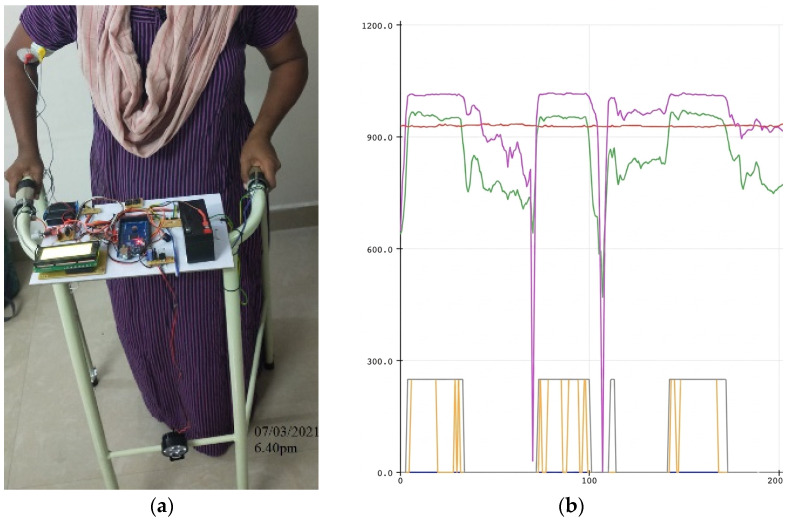
Gait Pattern and Posture Assessment on Day 7: (**a**) Subject 1’s mid-study posture shows improved upper-body alignment; (**b**) FSR trace demonstrates enhanced symmetry with FSI of 0.752.

**Figure 7:**
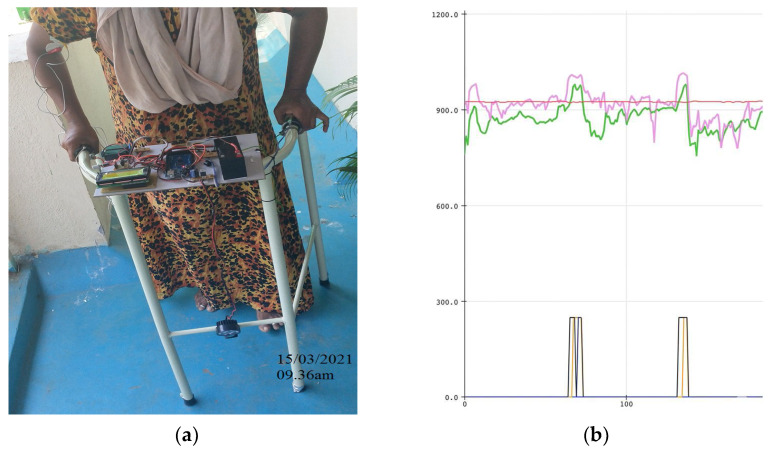
Gait Pattern and Posture Assessment on Day 15: (**a**) Subject 1’s posture at the end of the intervention, with markedly improved upright stance; (**b**) Force distribution shows near-symmetric patterns with consistent FSR signal morphology with FSI of 0.153.

Postural and Force Pattern Evolution (Subject 1 Case Study): Figure 5, Figure 6 and Figure 7 illustrate the progression of gait posture (subfigures a) and force distribution (subfigures b) for a representative participant (Subject 1). At baseline (Day 1), the subject exhibited pronounced asymmetry, with the majority of upper-limb support applied through one arm (Figure 5). By Day 7, partial restoration of postural alignment and improved force symmetry were observed (Figure 6). By Day 15, the subject demonstrated near-symmetric loading and an improved upright walking posture (Figure 7). This progression is also clearly visible in subfigure b of Figure 5, Figure 6 and Figure 7, where the color-coded lines in the plots correspond to the definitions provided in Figure 2.

**Figure 8:**
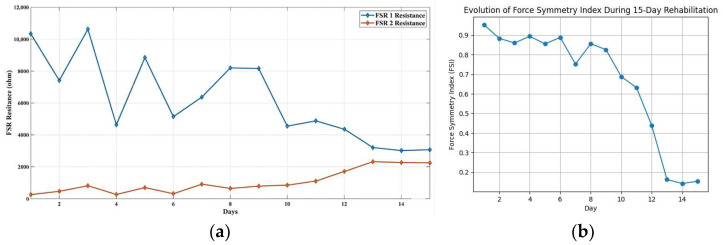
Quantitative Analysis of Force Symmetry Improvement. (**a**) Longitudinal FSR resistance measurements from the right (FSR1) and left (FSR2) walker handles across the 15-day rehabilitation period. The progressive convergence of the two signals indicates improved bilateral load distribution. (**b**) Subject 1’s Force Symmetry Index (FSI) calculated using the Robinson symmetry metric. The index decreased from 0.953 on Day 1 to 0.153 on Day 15, corresponding to an 83.9% reduction in load asymmetry, demonstrating effective motor adaptation during walker-assisted rehabilitation.

Longitudinal FSR resistance values for Subject 1 are shown in Figure 8a. Over the course of therapy, the resistance values of the two sensors gradually converged, indicating progressive redistribution of upper-limb support. At baseline: ${FSI}_{Day\;1}=0.953$, indicating severe asymmetry. Following the intervention: ${FSI}_{Day\;15}=0.153$, representing an 83.9% reduction in force asymmetry for subject 1. The temporal evolution of FSI values across the intervention period is shown in Figure 8b, demonstrating a consistent decline in asymmetry and confirming the effectiveness of the vibrotactile feedback mechanism. Supplementary Figure S7 illustrates the daily posture progression, highlighting a shift toward balanced arm force application and a significant improvement in force symmetry by Day 15 that correlates with restored posture.

### 3.2. A Algorithm Results: Predictive Modeling Using GPR

**Figure 9:**
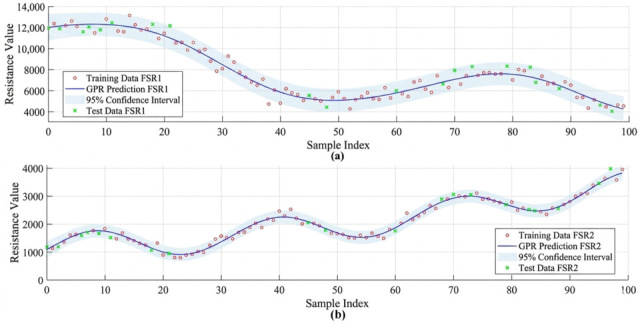
GPR-Based Prediction of FSR Resistance Trends. Gaussian Process Regression models applied to longitudinal FSR data: (**a**) GPR prediction curve for FSR1 shows high-fidelity fit with confidence bounds; (**b**) GPR prediction for FSR2 similarly tracks resistance trends with smooth, probabilistic estimation, indicating adaptive learning of rehabilitation progress.

To enable predictive monitoring of rehabilitation progress, a Gaussian Process Regression (GPR) model was trained on pooled longitudinal FSR resistance data collected over a 15-day period (Figure 9). The dataset consisted of 1200 time-series measurements across 10 participants, capturing variability in rehabilitation patterns. Model performance was evaluated using a held-out test set and 5-fold cross-validation. Predictive accuracy was quantified using the coefficient of determination (R^2^) and root mean squared error (RMSE). The model achieved R^2^ = 0.9604 for FSR1 and R^2^ = 0.9836 for FSR2, with RMSE values of 0.045 and 0.032, respectively, indicating strong agreement between predicted and observed values. The predicted trajectories, along with 95% confidence intervals, closely follow the observed data, demonstrating reliable uncertainty quantification. The model also showed robustness to missing data by interpolating trends from neighbouring observations. These results indicate strong predictive performance within the collected dataset and highlight the potential of the proposed framework for continuous rehabilitation monitoring.

### 3.3. Muscle Strength Analysis

**Figure 10:**
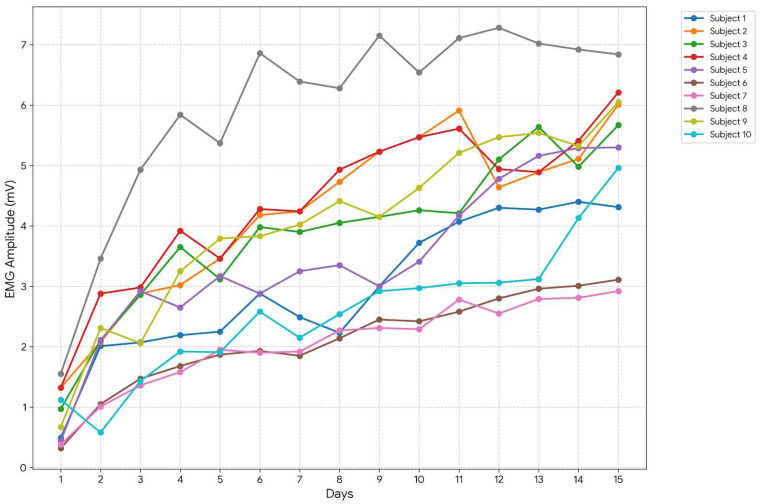
Group-Level Surface EMG Amplitude Progression: Mean sEMG signal amplitude across all 10 subjects over the 15-day period. The steady increase suggests improved neuromuscular engagement as reflected by increased muscle activation amplitude during assisted gait.

The group-level neuromuscular engagement was evaluated using surface EMG signals recorded during walker-assisted locomotion. Figure 10 illustrates the evolution of mean EMG amplitude across all participants during the 15-day intervention. At baseline (Day 1), the mean EMG amplitude across subjects was 0.885 mV. By the end of the rehabilitation program (Day 15), the mean EMG amplitude increased to 5.138 mV, reflecting a substantial increase in neuromuscular engagement during walker-assisted gait. The absolute change in muscle activation was therefore ΔEMG = 4.28 mV. To ensure the accuracy of these results, several preprocessing steps were applied to the raw EMG signals before analysis. A 4th-order digital band-pass filter was used to eliminate low-frequency drift and high-frequency noise, followed by digital wavelet denoising to further clean the signal. These preprocessing steps ensured signal reliability and the validity of the reported muscle activation results.

A paired *t*-test comparing Day 1 and Day 15 EMG amplitudes across the ten subjects yielded t(9) = 13.58, *p* < 0.001, indicating a statistically significant increase in neuromuscular activity. Prior to statistical analysis, the distribution of the data was assessed using histograms and Q–Q plots, which indicated approximate normality. Such visual methods are commonly used for preliminary assessment of distributional assumptions [62]. Furthermore, the paired *t*-test is known to be robust to moderate deviations from normality, particularly for small sample sizes and symmetric distributions [63]. Nevertheless, future studies will incorporate formal normality tests, such as the Shapiro–Wilk test and consider non-parametric alternatives where appropriate to further enhance statistical rigor.

### 3.4. Group-Wide Gait Symmetry Analysis

**Figure 11:**
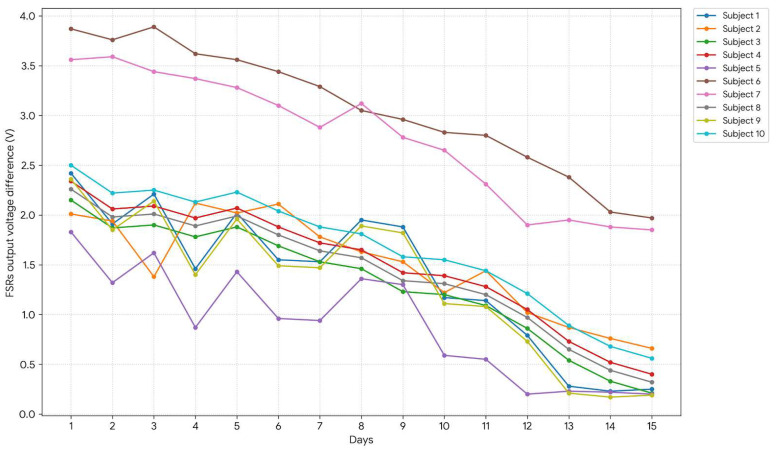
Interlimb Force Symmetry Improvement Over Time: Average voltage differential between FSR1 and FSR2 signals across participants, plotted over 15 days. Declining values indicate convergence in limb loading patterns and reduced compensatory asymmetry.

Figure 11 depicts the mean voltage difference between FSRs (left vs. right) for all subjects. A uniform reduction in inter-arm asymmetry over time is observed, affirming the walker’s role in enforcing bilateral coordination and reinforcing the findings from Subject 1’s case study. Although elderly participants (Subjects 6 and 7) exhibited slower improvement rates, all subjects demonstrated measurable progress in both neuromuscular engagement and load distribution.

**Figure 12:**
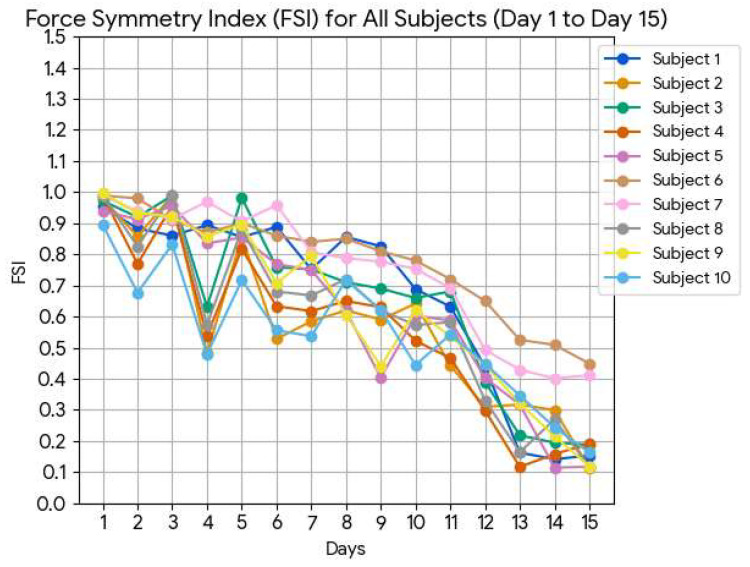
Progression of Force Symmetry Index (FSI) over the 15-day period for all 10 subjects, illustrating individual trends and variability in response to the intervention.

To provide a more comprehensive evaluation, group-level statistics were computed across all 10 subjects. The average Force Symmetry Index (FSI) on Day 1 was 0.9691, indicating a high degree of asymmetry at baseline. By Day 15, the average FSI decreased to 0.2019, reflecting a substantial improvement toward symmetrical force distribution over the intervention period. This corresponds to an average improvement of 79.26%, demonstrating a marked reduction in asymmetry across the cohort. To further illustrate inter-subject variability, Table 4 presents the Day 1 FSI, Day 15 FSI, and percentage improvement for each subject. Additionally, the temporal progression of FSI values over the 15-day period for all subjects is shown in Figure 12, highlighting both consistent trends and individual differences in response to the intervention. Before analysis, visual inspection of the FSI data distributions suggested approximate normality based on histograms and Q-Q plots [62]. Future analyses could formally test for normality to justify the use of parametric tests or, if necessary, consider non-parametric alternatives.

### 3.5. External Dataset Validation of Gait Variability Analysis (ALS Dataset)

**Figure 13:**
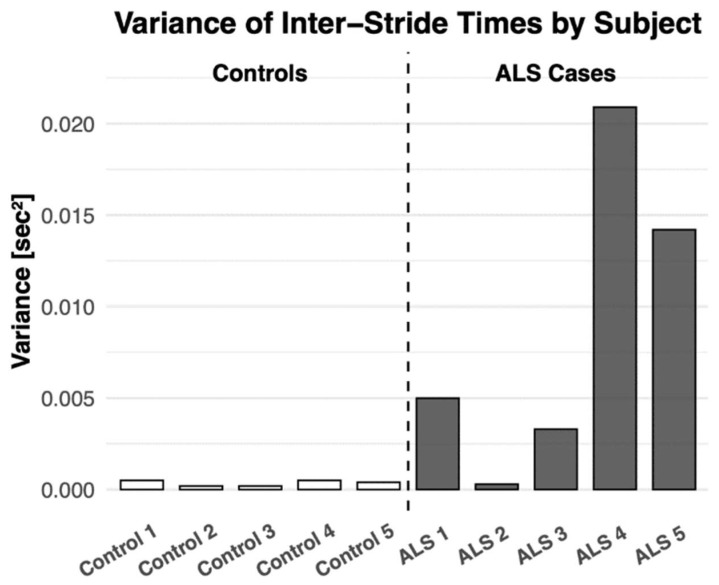
Variance in Inter-Stride Time Across ALS and Control Subjects: Bar chart comparing stride time variance in ALS patients versus healthy controls based on MATLAB-based gait cycle segmentation. Significantly elevated gait variability in the ALS group illustrates disrupted motor control, validating the walker’s potential for complex neuromuscular conditions.

To evaluate the generalizability of the analytical framework, an external dataset of gait recordings from patients with amyotrophic lateral sclerosis (ALS) was analyzed using the same MATLAB (R2025b)signal-processing pipeline [64]. Gait cycles were segmented using peak detection and transient filtering, allowing computation of stride-to-stride temporal variability. The resulting analysis revealed significantly higher stride time variance in ALS patients compared with healthy control subjects, as shown in Figure 13. This elevated variability is consistent with previously reported disruptions in corticospinal motor control associated with ALS and supports the use of temporal force dynamics as biomarkers of neuromuscular instability. Although this dataset was independent of the walker hardware, the findings demonstrate that the analytical framework developed in this work can effectively characterize gait variability patterns associated with neurological disorders. This suggests that the proposed system may eventually serve not only as a rehabilitation device but also as a platform for gait phenotyping and early detection of neuromotor dysfunction.

## 4. Discussion

Despite rapid advances in rehabilitation technology, several barriers continue to limit the widespread clinical adoption of digital gait monitoring systems. One major challenge is the reliance on large annotated datasets required for supervised machine learning models [22]. For example, markerless video-based gait analysis frameworks often depend on extensive labeled training data and convolutional neural networks (CNNs), which may struggle to generalize beyond homogeneous training populations. This limitation has been reported in several studies where models trained under controlled laboratory conditions fail to capture the variability of real-world gait patterns. In addition, the “black-box” nature of many deep learning algorithms reduces clinician trust and complicates clinical interpretation, while interoperability with healthcare systems—including electronic health records (EHRs), insurance frameworks, and clinical workflows—remains limited [22,23,24]. Similar challenges of generalizability and model transparency have also been observed in neural-network-based daily-life gait recognition using inertial sensors positioned at the lower back [65].

The SRW platform presented in this study was designed to address several of these limitations by prioritizing explainability, multimodal sensing, and home-based deployment. Rather than relying exclusively on opaque deep learning models, the system integrates interpretable signal processing with probabilistic modeling using Gaussian Process Regression (GPR). This approach allows clinicians to visualize rehabilitation trajectories while maintaining transparency in prediction uncertainty. The experimental results demonstrated substantial improvements in gait symmetry and neuromuscular engagement over the 15-day intervention period. The Force Symmetry Index (FSI) analysis revealed a significant reduction in inter-limb loading asymmetry, with values decreasing from severe asymmetry at baseline to substantially improved symmetry by the end of the rehabilitation period (FSI reduction from 0.953 to 0.153 for subject 1). Concurrently, group-level EMG analysis showed a marked increase in neuromuscular activation (ΔEMG ≈ 4.28), with the change reaching strong statistical significance (paired *t*-test, *p* < 0.001). These findings suggest that the vibrotactile feedback mechanism not only improves biomechanical symmetry but may also stimulate increased muscular engagement during assisted locomotion.

From a clinical perspective, the observed improvements in force symmetry have important implications for overall gait performance. Although the present system does not directly measure lower-limb kinematics, balanced upper-limb loading during walker-assisted ambulation is strongly associated with improved postural control and more symmetrical weight transfer between limbs. Previous studies have shown that asymmetrical loading is directly linked to impaired gait ability and increased fall risk [18], while interventions targeting load symmetry can influence gait symmetry and spatiotemporal coordination [19]. This relationship is particularly relevant in individuals with unilateral impairments, where excessive reliance on one side reinforces maladaptive gait patterns. By reducing force asymmetry, the proposed system encourages more uniform load distribution and may contribute to improved gait symmetry, reduced compensatory strategies, and enhanced walking efficiency. These findings support the use of upper-limb force symmetry as a meaningful and clinically relevant indicator in rehabilitation settings, particularly for continuous monitoring in home-based environments.

Collectively, the pilot study demonstrates that closed-loop multimodal feedback can promote measurable functional improvements in gait symmetry and neuromuscular activation. Nevertheless, several limitations must be acknowledged. The pilot cohort was relatively small and included heterogeneous gait pathologies. Larger cohort studies with stratified patient populations will be necessary to confirm the statistical robustness of the observed improvements. In addition, ergonomic refinements are required to optimize the walker for pediatric users and advanced geriatric populations. Finally, secure IoT integration and interoperability with clinical healthcare systems remain areas for future development.

Despite these limitations, the results suggest that the proposed system represents an important step toward integrated rehabilitation platforms that combine assistive mobility, physiological monitoring, and predictive analytics. With further validation, such platforms may evolve beyond assistive devices into comprehensive tools for long-term gait monitoring, personalized rehabilitation, and early detection of neuromuscular disorders.

## 5. Conclusions

This study presents a smart sensor–driven gait rehabilitation walker that integrates multimodal sensing, real-time haptic biofeedback, and explainable predictive modeling within a portable, home-ready platform. Unlike systems focused solely on measurement or cueing, the proposed framework unifies gait assessment, adaptive correction, and probabilistic forecasting within a closed-loop rehabilitation architecture. The 15-day pilot study demonstrated quantifiable improvements in gait symmetry and neuromuscular engagement. The Force Symmetry Index (FSI), computed using the Robinson symmetry metric, decreased from 0.953 at baseline to 0.153 after 15 days for subject 1, corresponding to an 83.9% reduction in inter-limb load asymmetry and an average of 0.9691 to 0.2019, corresponding to a 79.26% average reduction in inter-limb load asymmetry for all subjects. In parallel, surface electromyography measurements revealed a substantial increase in muscle activation (ΔEMG = 4.28), with statistical analysis confirming a significant improvement across participants (paired *t*-test: t(9) = 13.58, *p* < 0.001). These findings indicate that the walker’s haptic feedback mechanism effectively promotes balanced load distribution and enhanced neuromuscular engagement during assisted gait. Gaussian Process Regression further enabled accurate and uncertainty-aware prediction of rehabilitation trajectories from force sensor data, supporting individualized therapy progression. External dataset validation using ALS gait recordings confirmed the transferability of the analytical framework to neurodegenerative gait conditions beyond the initial rheumatoid arthritis cohort. By combining adaptive feedback, interpretable artificial intelligence, and scalable hardware integration, the proposed SRW functions as an intelligent biomechatronic co-therapist capable of measurement, prediction, and real-time rehabilitation intervention. This work bridges the gap between laboratory-grade gait analytics and accessible real-world rehabilitation technologies, laying the groundwork for decentralized, data-driven physiotherapy ecosystems and long-term gait health monitoring.

## Figures and Tables

**Table 1:** Comparative Analysis of Gait Detection and Monitoring Approaches.

Approach	Data Modality	Key Technologies	Strength	Limitations
Markerless Vision [23]	RGB video	Deep CNN, Self-Supervised Learning	Scalable, non-invasive	Sensitive to lighting, occlusions, dataset needs
Marker-Based Vision [24]	Motion capture	3D Skeletal Modeling	High precision, biomechanical richness	Expensive, lab-bound, labor-intensive
IMU Sensors [25]	Kinematics	SVM, Decision Trees	Wearable, suitable for field use	Sensor alignment sensitivity
EMG Sensors [26]	Myoelectric	LSTM, BiLSTM	Muscle activation insights	Susceptible to motion artifacts, skin noise
Structural Vibration [27,28]	Floor Sensors	FFT, Frequency Analysis	Passive, contactless	Requires custom infrastructure, sensitive to ambient noise
Hybrid GAN Models [29]	Multimodal and Synthetic	ConvLSTM, GAN	Data augmentation for rare disorders	Computationally complex, synthetic realism issues
Proposed Smart Rehabilitation Walker	FSR, sEMG, IMU, Haptic Feedback	Sensor Fusion, GPR Modeling	Real-time feedback, portable design, predictive monitoring	Requires initial calibration; easily resolved in future updates

**Table 2:** Summary of advantages and limitations of recent studies to improve gait rehabilitation and monitoring studies compared to the present work.

Study & Year	Method/Device	Advantages	Limitations
Zhang et al. [30]	IMU + plantar force sensors under treadmill conditions	High-accuracy fatigue detection in older adults; robust under controlled settings	Limited to single-condition treadmill use; no applicability to multi-morbidity or free-living monitoring
Cleland et al. [9]	Wearable sensors for post-stroke gait analysis	Identified predictors of walking speed and endurance	No real-time corrective feedback; lacks intervention capability
Khiyara et al. [31]	Haptic cueing to upper limbs	Demonstrated importance of arm–leg coupling for gait control	No neuromuscular monitoring; limited feedback modalities
Yentes et al. [32]	Gait time-series complexity analysis	Emphasized transparency and methodological rigor	Computational complexity; limited clinical translation
Franck et al. [33]	Bayesian statistical gait modeling	Quantifies uncertainty; informs predictor selection	Requires specialized expertise; high computational cost
Lee et al. [34]	Powered orthosis + kinematic modeling	Predicts rectus femoris hyperreflexia; accurate biomechanical modeling	Relies on precise kinematic simulations; less practical for home use
Present work	Smart rehabilitation walker with EMG, IMU, FSR, and haptic feedback	Multimodal monitoring, real-time corrective feedback, interpretable predictive modeling, home-based deployment	Requires validation in larger cohorts

**Table 3:** Clinical and demographic profile of study participants.

Subject	Age	Gender	Height (cm)	Weight (kg)	Diagnosis
Subject 1	50	F	158	65	RA with pronounced neurological gait impairments
Subject 2	47	F	155	54	RA with mild neurological gait features
Subject 3	44	F	158	67	RA with mild neurological gait features
Subject 4	48	F	168	58	RA with mild neurological gait features
Subject 5	51	M	158	72	RA with mild neurological gait features
Subject 6	68	M	165	66	RA
Subject 7	69	F	155	57	RA with mild neurological gait features
Subject 8	45	M	168	69	RA with pronounced neurological gait impairments
Subject 9	51	F	149	70	RA
Subject 10	55	F	152	53	RA with mild neurological gait features

**Table 4:** Force Symmetry Index (FSI) values on Day 1 and Day 15, along with percentage improvement for all subjects.

Subject	Day 1 FSI	Day 15 FSI	Improvement %
Subject 1	0.9526	0.1530	83.94%
Subject 2	0.9800	0.1129	88.48%
Subject 3	0.9721	0.1877	80.69%
Subject 4	0.9890	0.1905	80.74%
Subject 5	0.9390	0.1171	87.53%
Subject 6	0.9890	0.4489	54.61%
Subject 7	0.9890	0.4116	58.38%
Subject 8	0.9890	0.1157	88.30%
Subject 9	0.9962	0.1183	88.12%
Subject 10	0.8949	0.1630	81.79%

**Figure A1:**
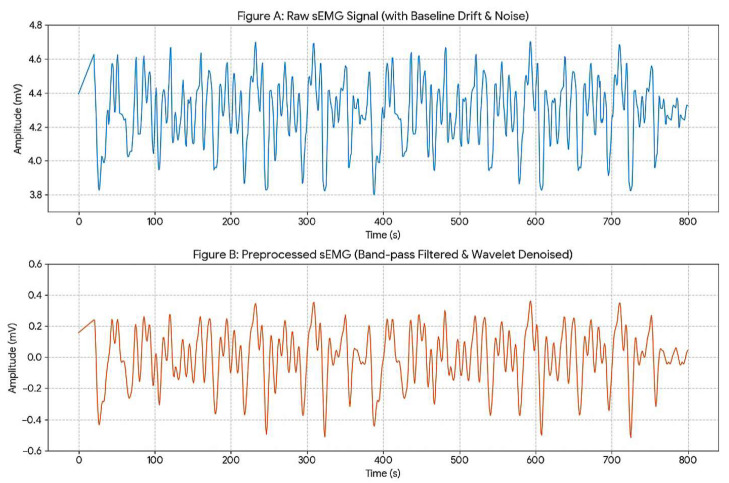
Illustration of the preprocessing stages applied to the raw surface electromyography (sEMG) signal.

## Data Availability

The datasets used and analyzed during the current study are available from the corresponding author on reasonable request.

## Supplementary Materials

The following supporting information can be downloaded at: https://www.mdpi.com/article/10.3390/s26082547/s1. Further details relevant to the study can be found in Supplementary Materials. Figure S1: Design Schematic of the Proposed Smart rehabilitation Walker: Orthographic design sketch showing front, side, and top views with dimensional annotations (in cm). This layout illustrates ergonomic planning and spatial allocation; Figure S2: Annotated Module Assembly of the Embedded Smart Rehabilitation Walker: Labeled breakdown of the integrated electronic and sensing subsystems: (1) LCD display, (2) EMG sensor, (3) triaxial accelerometer, (4) 9V battery for microcontroller, (5) dual FSRs, (6) Arduino Mega board, (7) Bluetooth module, (8) surface EMG electrodes, and (9) 12V power supply for haptic motors; Figure S3: sEMG Electrode Placement and Signal Acquisition Setup: Electrodes were positioned over the biceps brachii muscle to monitor upper limb muscular engagement. The reference electrode was placed near the lateral epicondyle of the humerus, following SENIAM guidelines for signal quality optimization; Figure S4: Real-time system output displayed via serial monitor over Bluetooth (wireless monitoring interface); Figure S5: System output rendered on the onboard LCD display (wired subject-side interface); Figure S6: Activation of the light assistance module under dark ambient conditions, demonstrating its utility in low-visibility environments; Figure S7: Temporal Progression of Walking Posture—Subject 1 Over 15 Days: Visual documentation of Subject 1’s posture during daily walking sessions across the 15-day study period, evidencing progressive improvements in gait symmetry and postural alignment.

## Appendix A

### Protocol for Patient Screening, Assessment, and Rehabilitation

To ensure systematic, reproducible, and ethically compliant evaluation, a study-specific protocol was developed in alignment with the capabilities of the proposed Smart Rehabilitation Walker. The protocol governed participant screening, gait assessment, assistive intervention, and data acquisition throughout the study.

A. Patient Screening

During initial consultations or routine follow-up visits, participants were screened using a structured questionnaire designed to identify functional gait instability. A key screening item was: “*Have you experienced a fall within the past three months?*”

Participants reporting recent falls were flagged for further clinical evaluation, while those without recent incidents proceeded to routine assessment and passive monitoring. This approach enabled efficient triaging of individuals with elevated fall risk.

B. Clinical Evaluation

Flagged individuals underwent a clinical evaluation conducted by a licensed physician, focusing on posture, dynamic balance, and lower-limb locomotor function. Observable features such as gait asymmetry, instability, reduced foot clearance, or compensatory movement patterns were documented. Individuals exhibiting functional gait impairments were referred for quantitative gait assessment using the Smart Rehabilitation Walker platform.

C. Quantitative Gait Assessment

Eligible participants underwent quantitative gait analysis using the sensor-integrated Smart Rehabilitation Walker. The system recorded and evaluated multiple gait parameters, including:

- Gait symmetry indices,
- Postural alignment,
- Surface electromyography (sEMG) of upper limb muscles,
- Tri-axial inertial sensor outputs (accelerometer and gyroscope),
- Ground-contact forces using force-sensing resistors (FSRs).

These data were used to characterize individual gait phenotypes and to establish baseline profiles for subsequent assistive intervention.

D. Smart Walker–Assisted Rehabilitation

This section provides a structured summary of the Smart Walker–assisted rehabilitation protocol. Detailed descriptions of the experimental setup, sensing modules, and rehabilitation methodology are provided in Section 2.2, Section 2.3 and Section 2.4 of the Methods section.

Participants exhibiting confirmed functional gait abnormalities were enrolled in a structured assistive walking program utilizing the modular Smart Rehabilitation Walker. The intervention emphasized voluntary gait execution supported by real-time feedback, rather than externally driven motion. Key components included:

- Real-time haptic feedback delivered via vibration motors to promote symmetric upper-limb loading and postural correction,
- Continuous inertial monitoring for fall detection and instability alerts,
- Ambient lighting assistance to enhance navigation safety in low-illumination environments,
- sEMG monitoring of the biceps brachii to track neuromuscular engagement during assisted ambulation.

Walking sessions (approximately 80 m per day) were conducted either under direct physiotherapist supervision in a clinical environment or at home with remote monitoring, depending on participant capability and safety considerations. Predictive modeling using Gaussian Process Regression (GPR) was applied to FSR data to evaluate rehabilitation trends and responsiveness over time. All procedures were conducted in accordance with institutional ethical guidelines and the principles of the Declaration of Helsinki. Ethical approval was obtained from the Institutional Ethics Committee of Rajalakshmi Engineering College, India. Written informed consent was obtained from all participants prior to study participation.

## Appendix B

Figure A1 illustrates the preprocessing stages applied to the raw surface electromyography (sEMG) signal. The raw signal (Figure A1A) contains the desired muscle activity components along with significant baseline drift and high-frequency noise. The baseline drift is primarily attributed to electrode motion, skin impedance variations, and environmental factors, while the noise includes power-line interference and other measurement artifacts. As a result, the raw signal is not directly suitable for further analysis. To address these issues, a two-stage preprocessing pipeline was employed. First, a band-pass filter was applied to remove low-frequency baseline drift and high-frequency noise, preserving only the frequency components relevant to muscle activity. This step effectively centers the signal around zero and enhances its interpretability. Subsequently, wavelet-based denoising was performed to further suppress residual noise while preserving important signal features such as transient peaks associated with muscle activation. Compared to conventional filtering techniques, wavelet denoising provides improved time–frequency localization, enabling more effective noise reduction without distorting the underlying signal structure. The resulting preprocessed signal (Figure A1B) exhibits significantly reduced noise and drift, with enhanced clarity of the underlying muscle activity. This processed signal serves as a reliable input for subsequent feature extraction and analysis.

## Appendix C

**Table A1:** Summary of Gaussian Process Regression model dataset, training/validation, and predictive performance metrics for FSR1 and FSR2.

Feature/Metric	FSR1	FSR2	Description/Notes
Number of participants	10	10	Separate cohort for each FSR channel
Total time-series measurements	1200	1200	Number of data points collected per FSR channel over 15 days
Unit of measurement	Samples	Samples	Each sample corresponds to a single sensor reading at a time index
Input variable	Time index (x)	Time index (x)	Represents time of sensor measurement
Output variable	FSR resistance (y)	FSR resistance (y)	Represents measured handlebar force
Training dataset	80% of total samples	80% of total samples	Randomly selected pooled data for model training
Test dataset	20% of total samples	20% of total samples	Held-out data for evaluating generalization
Cross-validation	5-fold	5-fold	Performed on training data to optimize hyperparameters
Kernel type	RBF	RBF	Assumes smooth function behavior
Hyperparameter optimization	Log marginal likelihood	Log marginal likelihood	Optimized signal variance, length-scale, and noise variance
Predictive performance (R^2^)	0.9604	0.9836	Measures fit of predicted vs. observed
RMSE	0.045	0.032	Quantifies prediction error
Confidence intervals	95%	95%	Captures uncertainty of predictions
