# Supplementary material for: Smart Sensor-Driven Gait Rehabilitation Walker Using Machine Learning for Predictive Home-Based Therapy
Source: Sensors (Basel). 2026 Apr 21;26(8):2547. doi: 10.3390/s26082547 (PMC13120546; doi:10.3390/s26082547)
Supplement: Supplementary file 1 [file sensors-26-02547-s001.zip › sensors-4223951-supplementary.pdf]

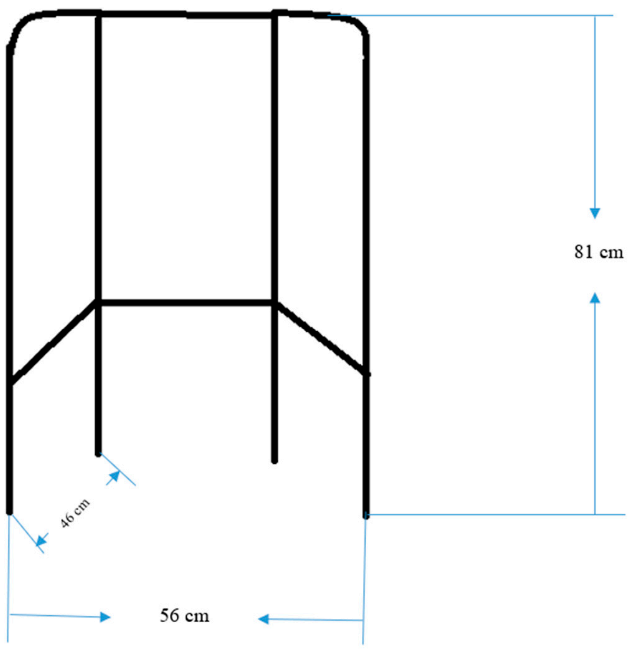

Figure S1. Design Schematic of the Proposed Smart rehabilitation Walker: Orthographic design sketch showing front, side, and top views with dimensional annotations (in cm). This layout illustrates ergonomic planning and spatial allocation.

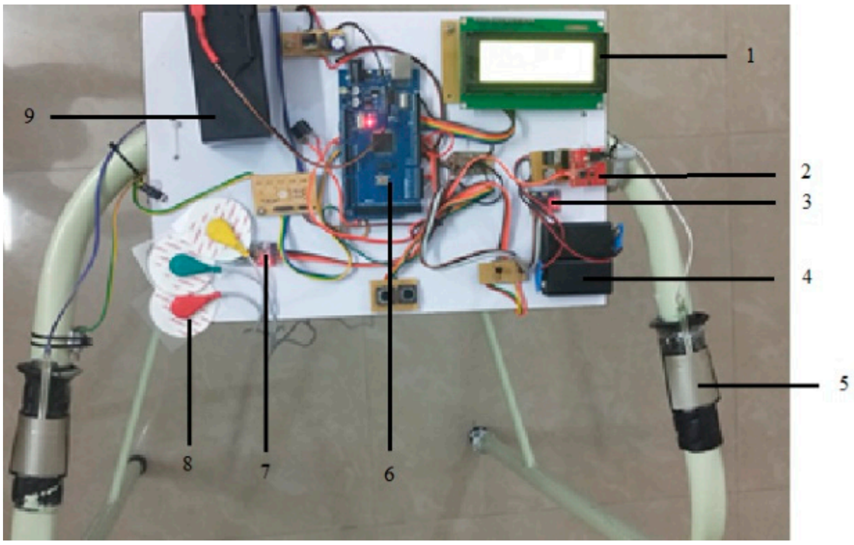

Figure S2. Annotated Module Assembly of the Embedded Smart Rehabilitation Walker: Labeled breakdown of the integrated electronic and sensing subsystems: (1) LCD display, (2)

EMG sensor, (3) triaxial accelerometer, (4) 9V battery for microcontroller, (5) dual FSRs, (6) Arduino Mega board, (7) Bluetooth module, (8) surface EMG electrodes, and (9) 12V power supply for haptic motors.

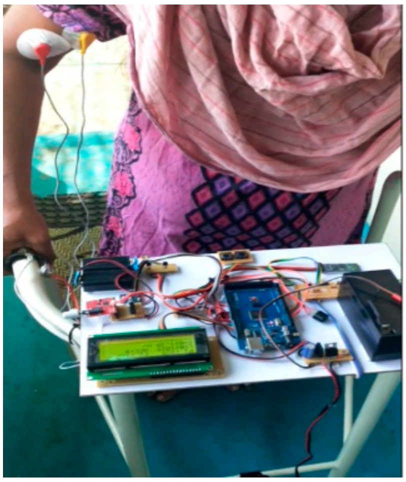

Figure S3: sEMG Electrode Placement and Signal Acquisition Setup: Electrodes were positioned over the biceps brachii muscle to monitor upper limb muscular engagement. The reference electrode was placed near the lateral epicondyle of the humerus, following SENIAM guidelines for signal quality optimization.

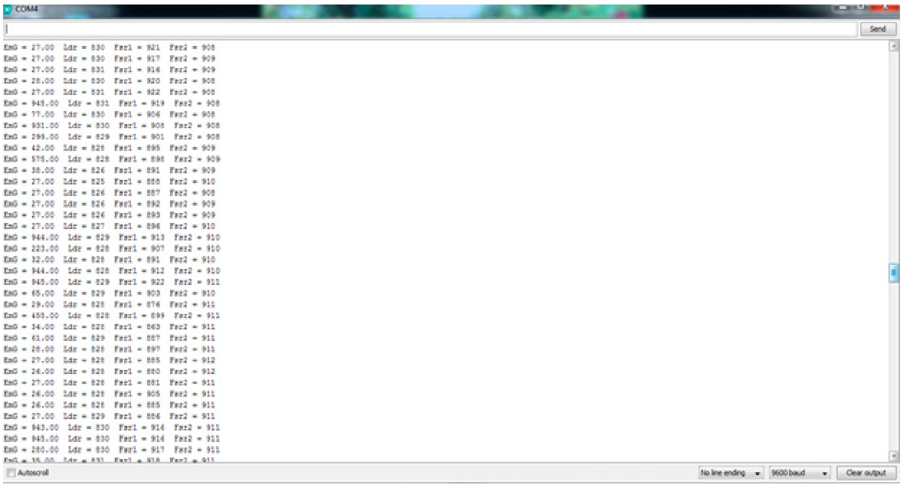

Figure S4. Real-time system output displayed via serial monitor over Bluetooth (wireless monitoring interface).

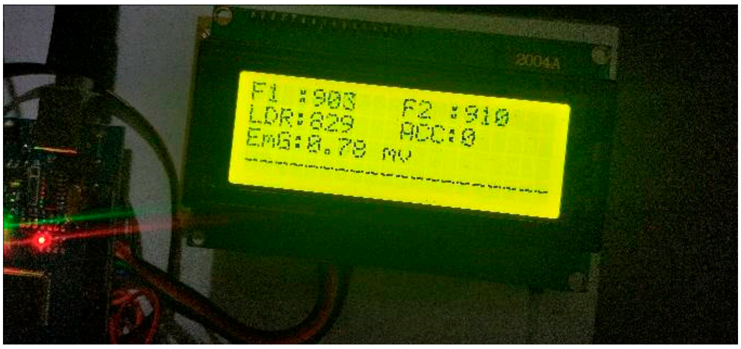

Figure S5. System output rendered on the onboard LCD display (wired subject-side interface).

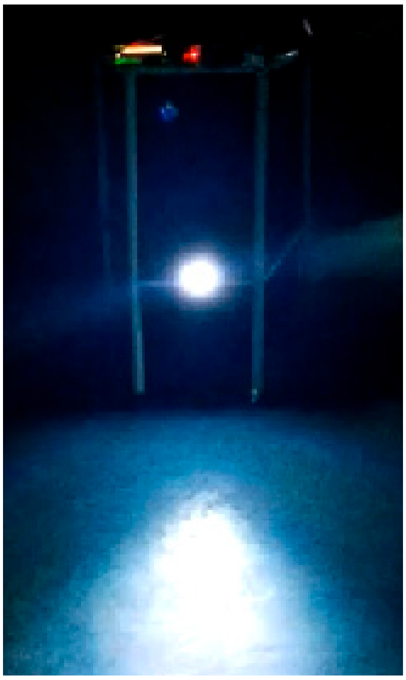

Figure S6. Activation of the light assistance module under dark ambient conditions, demonstrating its utility in low-visibility environments.

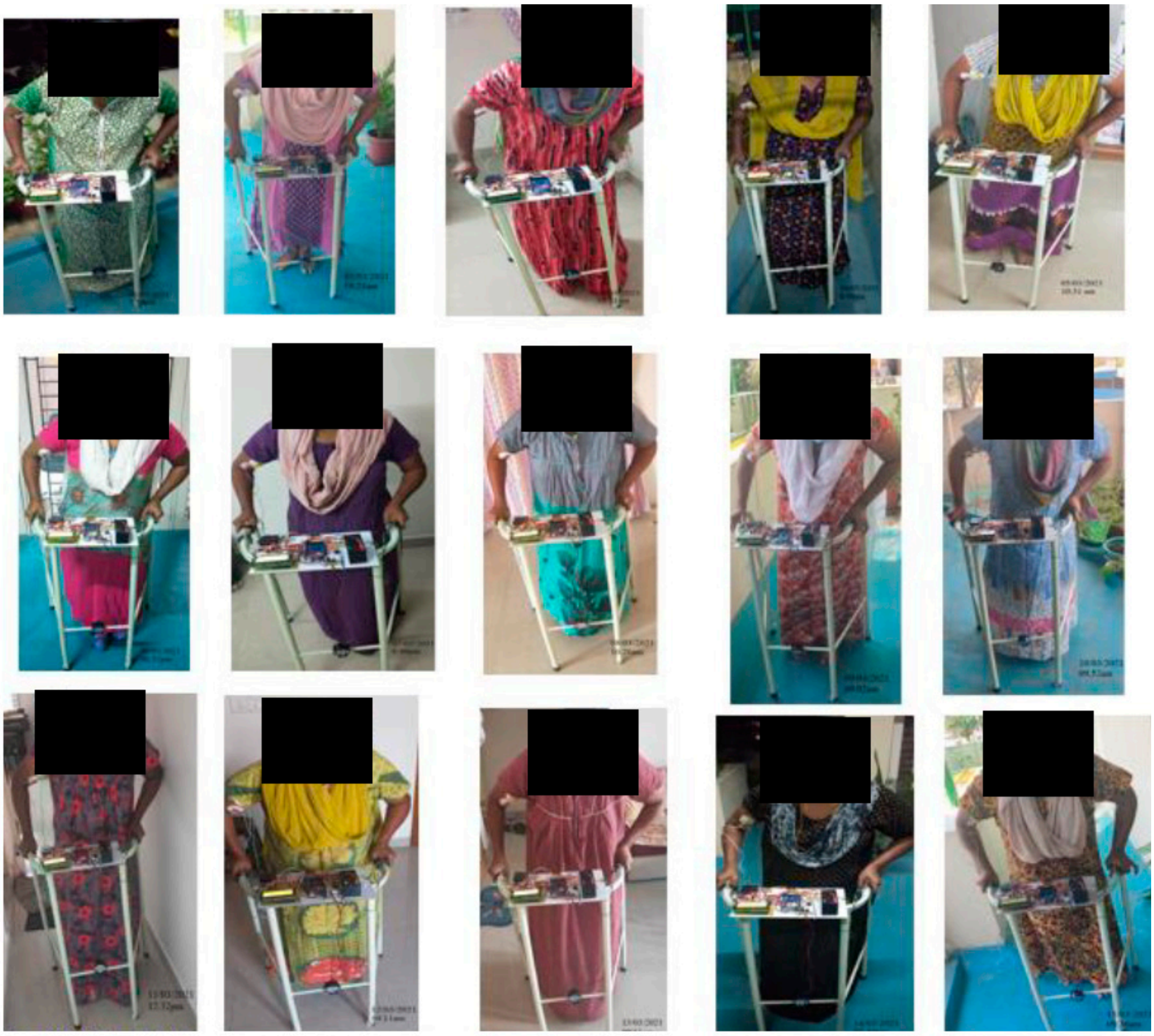

Figure S7: Temporal Progression of Walking Posture – Subject 1 Over 15 Days: Visual documentation of Subject 1's posture during daily walking sessions across the 15-day study period, evidencing progressive improvements in gait symmetry and postural alignment.
